# The Indications of Tonsillectomy Among Pediatric Patients: Our Nine-Year Retrospective Review

**DOI:** 10.7759/cureus.50638

**Published:** 2023-12-16

**Authors:** Essam S Alzahrani, Ibrahim A Aseeri, Waleed J Alzahrani, Mazen S Alharthi, Faris M Qattan, Mohammed Khan

**Affiliations:** 1 College of Medicine, Taif University, Taif, SAU; 2 Otolaryngology Head and Neck Surgery, King Abdullah Medical City, Makkah, SAU

**Keywords:** pediatrics, indications for surgery, tonsillitis, ent procedures, tonsillectomy

## Abstract

Introduction: Tonsillectomy is a surgical procedure that involves removing the tonsils, often performed alongside adenoidectomy. Dating back to ancient times, it improves quality of life and can be life-saving when done for appropriate reasons. Common indications in children include recurrent tonsillitis and sleep-disordered breathing (SDB). Evidence suggests it reduces how often and how severe sore throats are in highly affected children. Symptoms such as lymphadenopathy, tonsillar pus, fever, or signs of streptococcal infection should be present for diagnosis of tonsillitis. Polysomnography (PSG) is essential to diagnose obstructive sleep apnea (OSA) and confirm airway obstruction.

Aim: Our aim in this study is to determine the reasons for tonsillectomy in Taif, Saudi Arabia, since it is not well established before in this city.

Methods: A nine-year retrospective analysis of case records of patients aged 0-18 years who have had tonsillectomy performed in a tertiary hospital. Data was analyzed using SPSS (IBM Corp., Armonk, NY, USA).

Results: The research study involved analyzing data from 361 participants. Among the participants, 16.9% (n = 61) underwent tonsillectomy alone, while the majority (83.1%; n = 300) underwent tonsillectomy in combination with other procedures. The most common combined procedure was adenotonsillectomy (71.7%; n = 259). Additionally, adenotonsillectomy and insertion of grommets in other combinations was observed and was equal to 11.4% (n = 41) of the total percentage of our sample. The primary indications for tonsillectomy alone were chronic tonsillitis (42.6%; n = 26) and recurrent tonsillitis (49.2%; n = 30).

Conclusion: This research study provides valuable information on the types of procedures performed and the indications for surgery in pediatric patients. The results highlight the prevalence of chronic and recurrent tonsillitis and adenotonsillitis as primary indications for tonsillectomy, either alone or in combination with other procedures. These findings contribute to our understanding of the clinical decision-making process and can aid healthcare professionals in providing optimal care for pediatric patients with tonsillar and adenotonsillar pathologies.

## Introduction

Tonsillectomy is a surgical procedure that involves dissecting the peritonsillar gap between the tonsil capsule and the muscle wall to fully remove the tonsil, including its capsule. It can be done with or without adenoidectomy [1]. Tonsillectomy is an ancient surgery that goes back to the first century A.D. [2]. Tonsillectomy enhances quality of life and can even save lives when performed for the right indications [3]. Infections and blockages are the most prevalent indications for tonsillectomy and adenotonsillectomy across the world [2]. In children, tonsillectomy is still a popular treatment for treating recurrent tonsillitis and sleep-disordered breathing (SDB) including obstructive sleep apnea (OSA) [4]. Evidence suggests that tonsillectomy reduces recurring sore throats in highly afflicted children (ages three to 15 years), but not in less seriously affected children [5]. Patients who had more than seven bouts of tonsillitis in a year, five episodes in two years, or three occurrences in the previous three years showed a benefit [6]. At least one of the following symptoms should be present in a sore throat: lymphadenopathy, tonsillar pus, fever, or signs of streptococcal infection [5]. Snoring, mouth breathing, and breathing pauses are all symptoms of SDB that can be clinically diagnosed. The presence of blockage must be confirmed by polysomnography (PSG) in order to diagnose OSA [6]. With the fall of infection as a reason for surgery in children, detecting airway obstruction and SDB has become increasingly crucial, especially given the 4-11% prevalence of SDB reported by parents [7].

Tonsillectomy is a widely known surgical procedure, yet the indications of tonsillectomy are not well documented in Saudi Arabia, specifically in the city of Taif. The frequency of tonsillitis and tonsillectomy varies, and the medical system is failing to adjust to this variation. Also, the mismatch between health care guidelines and awareness of people about tonsillectomy currently has affected the type of indications that used to be considered for tonsillectomy. The aim of this study is to determine the reasons for tonsillectomy in Taif because of the absence of studies about this topic in this city.

## Materials and methods

This is a nine-year retrospective review of case data from patients aged 0 to 18 who had tonsillectomy in a tertiary hospital. The hospital is located in the southwest of Taif; it was chosen because it’s the only specialized hospital that serves the Taif population, plus those from the villages that belong to the governorate of Taif.

Sampling and population

The pool of participants in this study included the case files of any child ages 0 to 18 despite the parity of gender who underwent tonsillectomy in King Abdulaziz Specialist Hospital from 2014 to 2022.

The study took place at Taif, which is located in Saudi Arabia's western part, Makkah Province. Taif's population was estimated to be 986,916 [8].

A total of 361 samples were found to be fit for our criteria. We took the samples using an Excel file (Microsoft, Redmond, WA, USA) containing patient file numbers of our target patients.

The study was approved by the research ethics committee department of the Directorate of Health Affairs in Taif City (approval number: 744, date: 20.09.2022).

Inclusion criteria

All case files for children ages 0 to 18 who underwent tonsillectomy from 2014 to 2022. 

Exclusion criteria

Any child with co-morbid conditions, e.g., neoplastic or syndromic conditions, any child with incomplete case files missing important data and any case file for patients aged more than 18 years.

Tools and data collection procedure

We used a protected Excel file containing information about age, gender, date of procedure, procedure performed and indication of surgery, and any co-morbid conditions. We then reviewed the data from selected archival files for the years 2014-2022 inclusively in King Abdulaziz Specialist Hospital and excluded any file that didn’t meet the inclusion criteria, then we entered the data in a protected Excel file.

The conditions were classified and defined according to the ICD-10 which is approved and used by archives at the King Abdulaziz Specialized Hospital in Taif.

After checking the data for completeness and ensuring that it met the inclusion requirements, we coded and classified the proper data. The data was categorized depending on their age, gender and indication for surgery, after that, we entered the data into the SPSS software program version 27.0.1 (IBM Corp., Armonk, NY, USA). Then, using SPSS, we tabulated the findings and evaluated them graphically and statistically. To protect privacy, no personal information, including name or contact details, was collected and was only accessible to the authors. This study was self-funded.

Statistical analysis

After checking the data for completeness and ensuring that it meets the inclusion requirements, we coded and classified the proper data. Simple descriptive analysis of the data was carried out. Descriptive statistics of the sociodemographic characteristics and other categorical variables in the form of frequencies and percentages were calculated and tabulated. For continuous variables, namely the ages of the participants, means and standard deviations were calculated and reported as measures of central tendency and dispersion respectively. All statistical calculations were performed using SPSS version 27.0.1.

## Results

A total of 361 participants satisfying the inclusion criteria were included in the analysis, with an average age of 6.92 years (SD = 3.38). In terms of gender distribution, 150 participants were female, accounting for 41.6% of the total sample, while 211 participants were male, representing 58.4% of the sample (Table 1).

**Table 1:** Sociodemographic characteristics of the participants

Category		Average age / Number of cases	SD of age / Percentage of each category
Age (Years)		6.92	3.38
Gender	Female	150	41.6%
	Male	211	58.4%
Total		361	100.0%

Number of procedures

Table 2 provides an overview of the procedural frequencies observed in the study. Out of the total 361 participants, 61 individuals (16.9%) underwent tonsillectomy as a standalone procedure. The majority of participants, specifically 300 individuals (83.1%), underwent tonsillectomy combined with other procedures. Within this category of combined procedures, the most prevalent was adenotonsillectomy, which was performed in 259 cases (71.7%). Moreover, a combination of adenotonsillectomy along with the insertion of grommets was performed in 41 cases (11.4%).

**Table 2:** Frequency of each procedure done

Procedure		N	%
Tonsillectomy Alone		61	16.9%
Tonsillectomy in Combination with Other Procedures		300	83.1%
Combined with	Adenotonsillectomy	259	71.7%
	Adenotonsillectomy and Insertion of Grommets	41	11.4%

Indications for surgery

In the dataset presented in Table 3, we can observe the indications for both standalone tonsillectomy and tonsillectomy performed in conjunction with other procedures.

**Table 3:** Indications for tonsillectomy alone and tonsillectomy in combination with other procedures

Procedure	Indication	N	%
Tonsillectomy (N=61)	Recurrent Tonsillitis	30	49.2%
	Chronic Tonsillitis	26	42.6%
	Recurrent Adenotonsillitis	4	6.6%
	Chronic Adenotonsillitis	1	1.6%
Tonsillectomy In Combination with Other Procedures (N=300)			
Adenotonsillectomy (N= 259)	Chronic Adenotonsillitis	141	54.4%
	Recurrent Adenotonsillitis	113	43.6%
	Recurrent Tonsillitis	1	0.4%
	Chronic Tonsillitis	2	0.8%
	Chronic Adenotonsillar Hypertrophy	2	0.8%
Adenotonsillectomy And Insertion of Grommets (N=41)	Chronic Adenotonsillitis	32	78.0%
	Recurrent Adenotonsillitis	6	14.6%
	Chronic Tonsillitis	2	4.9%
	Adenoid Hypertrophy	1	2.4%

For the cases involving standalone tonsillectomy (N=61), the predominant indications were recurrent tonsillitis, accounting for 30 cases (49.2%), and chronic tonsillitis, accounting for 26 cases (42.6%). There were also a few instances of tonsillectomy carried out due to recurrent adenotonsillitis (four cases, 6.6%) and chronic adenotonsillitis (one case, 1.6%).

When considering tonsillectomy in combination with other procedures (N=300), specifically adenotonsillectomy (N=259), the primary indication was chronic adenotonsillitis, with 141 cases (54.4%), followed by recurrent adenotonsillitis, with 113 cases (43.6%). A minor number of cases exhibited indications such as recurrent tonsillitis (one case, 0.4%), chronic tonsillitis (two cases, 0.8%), and chronic adenotonsillar hypertrophy (two cases, 0.8%).

Additionally, when focusing on adenotonsillectomy combined with the insertion of grommets (N=41), the primary indication was chronic adenotonsillitis, with 32 cases (78.0%). Recurrent adenotonsillitis was also an indication in six cases (14.6%), while chronic tonsillitis and adenoid hypertrophy were cited as indications in two cases (4.9%) and one case (2.4%) respectively.

These findings highlight the noteworthy role of chronic and recurrent tonsillitis (42.6% (n = 26) and 49.2% (n = 30) respectively) and chronic and recurrent adenotonsillitis (54.4% (n = 141) and 43.6% (n = 113) respectively) as the leading indications for tonsillectomy, both when performed independently and in combination with other procedures (Table 3).

## Discussion

The aim of our study was to investigate the indications of tonsillectomy in our population and compare it to other studies. The analysis included a total of 361 participants, with an average age of 6.92 years. The gender distribution revealed a slight predominance of males, representing 58.4% of the sample.

The majority of participants underwent tonsillectomy, either alone or in combination with other procedures. Among the combined procedures, adenotonsillectomy was the most common, followed by adenotonsillectomy combined with the insertion of grommets. The finding that a significant proportion of participants underwent tonsillectomy alone (16.9%) suggests that tonsillectomy continues to be a frequently performed procedure. This finding aligns with previous studies that have also reported tonsillectomy as a common surgical intervention for conditions such as recurrent tonsillitis or OSA [9,10]. 

The most common combination observed in this study was adenotonsillectomy, accounting for 71.7% of the cases. This finding is consistent with previous research indicating that adenotonsillectomy is often performed for the treatment of concurrent adenotonsillar hypertrophy, a common condition in pediatric patients [11]. The high frequency of adenotonsillectomy in this study highlights the clinical significance of evaluating both the adenoids and the tonsils when managing pediatric patients with upper airway pathology.

Furthermore, the combination of adenotonsillectomy with the insertion of grommets was performed in 10.2% of cases. This procedure combination is often employed to address both adenotonsillar hypertrophy and otitis media with effusion, as grommets help to alleviate middle ear fluid accumulation [12,13]. The relatively low frequency of this combination procedure in comparison to adenotonsillectomy alone may reflect variations in patient presentation and otolaryngologists’ clinical decision-making processes.

In our study, among the cases of tonsillectomy alone, the most common indications were chronic tonsillitis (42.6%) and recurrent tonsillitis (49.2%). These findings are consistent with previous studies that have reported chronic and recurrent tonsillitis as the leading indications for tonsillectomy [5,14]. Chronic tonsillitis is characterized by persistent inflammation and infection of the tonsils, while recurrent tonsillitis refers to multiple episodes of acute tonsillitis within a defined period. These conditions can significantly impact the quality of life and overall health of patients, often leading to frequent sore throats, difficulty swallowing, and sleep disturbances.

Furthermore, our study examined cases where tonsillectomy was performed in combination with other procedures, such as adenotonsillectomy, grommet insertion, myringotomy, and adenectomy. The primary indication for tonsillectomy combined with other procedures was chronic adenotonsillitis and chronic tonsillitis for adenectomy. These findings highlight the significance of chronic adenotonsillitis as a common indication for combined surgical interventions [10]. Chronic adenotonsillitis, characterized by persistent inflammation of both the adenoids and tonsils, can lead to recurrent infections, OSA, and other complications [15]. Performing a combination of procedures allows for comprehensive management of these conditions and better outcomes for the patients.

It is important to acknowledge the limitations of this study. The data was obtained from a single center, which may limit the generalizability of the findings to other populations or healthcare settings. Additionally, the lack of polysomnography in our center and the lack of patients diagnosed with OSA in our records is another limitation. Future research could explore these aspects to further enhance our understanding of the management of tonsillar and adenotonsillar pathologies in pediatric patients.

## Conclusions

This research study provides valuable information on the types of procedures performed and the indications for surgery in pediatric patients. The results highlight the prevalence of chronic and recurrent tonsillitis and adenotonsillitis as primary indications for tonsillectomy and adenotonsillectomy, either alone or in combination with other procedures. These findings contribute to our understanding of the clinical decision-making process and can aid healthcare professionals in providing optimal care for pediatric patients with tonsillar and adenotonsillar pathologies.
